# New Approaches in Nanomedicine for Ischemic Stroke

**DOI:** 10.3390/pharmaceutics13050757

**Published:** 2021-05-20

**Authors:** Clara Correa-Paz, Andrés da Silva-Candal, Ester Polo, Jérôme Parcq, Denis Vivien, Dusica Maysinger, Beatriz Pelaz, Francisco Campos

**Affiliations:** 1 Clinical Neurosciences Research Laboratory (LINC), Health Research Institute of Santiago de Compostela (IDIS), 15706 Santiago de Compostela, Spain; clara.correa@hotmail.es; 2 Neurology Service, University Hospital Complex of A Coruña, A Coruña Biomedical Research Institute, 15006 A Coruña, Spain; andres.dasilva.candal@gmail.com; 3 Centro Singular de Investigación en Química Biolóxica e Materiais Moleculares (CiQUS), Universidade de Santiago de Compostela, 15782 Santiago, Spain; ester.polo@usc.es (E.P.); beatriz.pelaz@usc.es (B.P.); 4 Grupo de Física de Coloides y Polímeros, Departamento de Física de Partículas, Universidade de Santiago de Compostela, 15782 Santiago, Spain; 5 Op2Lysis SAS, 14000 Caen, France; jerome.parcq@op2lysis.com; 6 UNICAEN, INSERM U1237, Etablissement Français du Sang, Physiopathology and Imaging of Neurological Disorders (PhIND), Cyceron, Institut Blood and Brain @ Caen-Normandie (BB@C), Normandie University, 14000 Caen, France; vivien@cyceron.fr; 7 Department of clinical research, Caen-Normandie University Hospital, CHU, Avenue de la côte de Nacre, 14000 Caen, France; 8 Department of Pharmacology & Therapeutics, McGill University, Montréal, QC H3G 1Y6, Canada; dusica.maysinger@mcgill.ca; 9 Grupo de Física de Coloides y Polímeros, Departamento de Química Inorgánica, Universidade de Santiago de Compostela, 15782 Santiago, Spain

**Keywords:** cell tracking, diagnosis, drug carriers, drug control release, nanomedicine, neuroprotection, recovery, stroke, tissue plasminogen activator

## Abstract

Ischemic stroke, caused by the interruption of blood flow to the brain and subsequent neuronal death, represents one of the main causes of disability in developed countries. Therapeutic methods such as recanalization approaches, neuroprotective drugs, or recovery strategies have been widely developed to improve the patient’s outcome; however, important limitations such as a narrow therapeutic window, the ability to reach brain targets, or drug side effects constitute some of the main aspects that limit the clinical applicability of the current treatments. Nanotechnology has emerged as a promising tool to overcome many of these drug limitations and improve the efficacy of treatments for neurological diseases such as stroke. The use of nanoparticles as a contrast agent or as drug carriers to a specific target are some of the most common approaches developed in nanomedicine for stroke. Throughout this review, we have summarized our experience of using nanotechnology tools for the study of stroke and the search for novel therapies.

## 1. Introduction

### 1.1. Stroke

**Figure 1:**
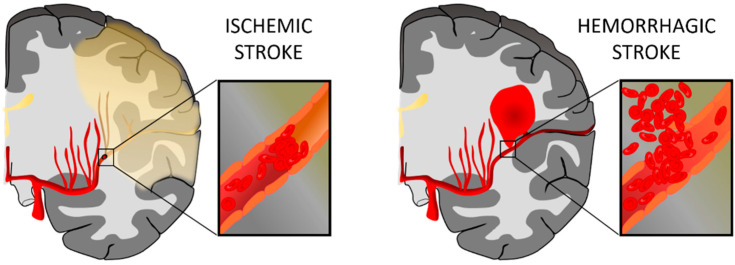
Representation of ischemic stroke and hemorrhagic stroke. In an ischemic stroke, the clot blocks blood flow to an area of the brain; meanwhile, hemorrhagic stroke happens when a blood vessel breaks and bleeds into the brain. Figure created with BioRender.com.

Stroke is a cerebrovascular disease resulting from disturbance in normal cerebral blood flow (CBF), which causes a transient or permanent deficit in the function of one or more parts of the brain, and is becoming one of the leading causes of disability in developed countries. The disturbance of normal CBF induces metabolic and cellular changes that can lead to cell death and disruption of the nervous system [1]. Stroke can be classified into two types: hemorrhagic and ischemic. A hemorrhagic stroke is due to a blood vessel rupture, represents up to 15% of all stroke cases, and is associated with high mortality. On the contrary, an ischemic stroke is caused by an obstruction in the vessel, often due to the presence of a clot, and constitutes approximately 85% of stroke events [2] (Figure 1).

**Figure 2:**
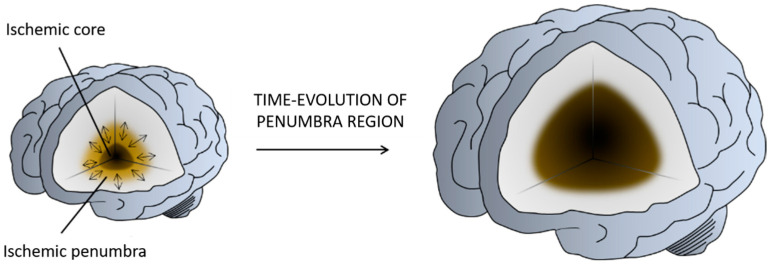
Illustration of the penumbra concept. Ischemic core represents the infarcted tissue and the penumbra region the salvageable tissue at risk for infarction in case of persistence vessel occlusion. While neurons in the ischemic core are considered beyond rescue, neurons in the penumbra are potential targets for therapeutic intervention. Figure created with BioRender.com.

The ischemic infarct is a dynamic region in which multiple molecular and cellular processes are involved (defined as an ischemic cascade), which starts immediately after the ischemic insult and changes even weeks and months later. Based on the blood perfusion level and metabolic activity in the infarct brain, two regions have been extensively described: the ischemic core and the penumbra [3] (Figure 2). The core is the region most affected by the lack of blood flow and is irreversibly injured in a few minutes. The penumbra is a vulnerable, hypo-perfused, and metabolically compromised region that can be recovered if the blood flow supply is re-established in the first few hours after a stroke. The penumbra is also the target of multiple protective drugs aimed at reducing or blocking the progression of cell death [4,5].

**Figure 3:**
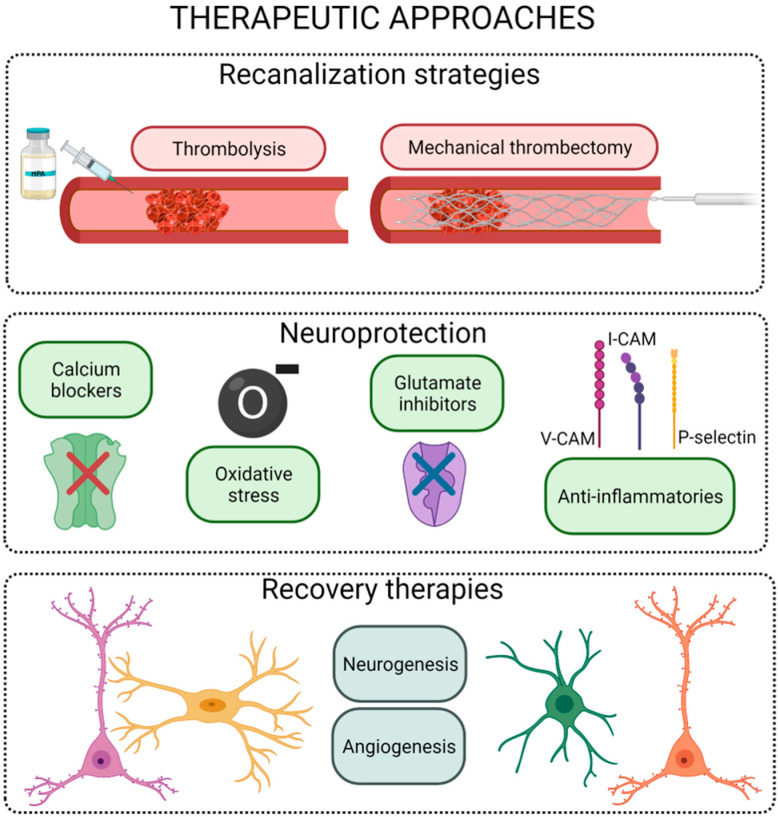
Scheme of the different therapeutic strategies for the ischemic stroke. Figure created with BioRender.com.

Therapeutic approaches after stroke onset may be classified into three main groups: reperfusion, protective, and recovery strategies (Figure 3).

Reperfusion therapies, based on the use of pharmacological thrombolysis and/or mechanical recanalization, are focused on restoring CBF in the first hours post stroke onset [6]. Thrombolysis with intravenous recombinant tissue plasminogen activator (rtPA) is the only Food and Drug Administration/European Medicines Agency (FDA/EMA)-approved drug treatment for patients with acute ischemic stroke, but its use is limited by a narrow therapeutic window, selective efficacy, and hemorrhagic complications [7]. Nowadays, advanced endovascular recanalization technology based on the use of mechanical catheters has significantly improved the recanalization rates, showing a better overall outcome in treated patients. However, this mechanical therapy is effective when stroke events are caused by a large vessel occlusion, which represents less than 30% of the patients. Moreover, in occlusions located in small vessels, where the rtPA has a successful recanalization rate, the efficacy of mechanical thrombectomy is also limited. In addition, some clinical trials have shown that pretreatment with intravenous rtPA provides additional benefits to patients undergoing mechanical thrombectomy. These evidences reflect that rtPA thrombolysis remains the gold standard treatment for acute stroke [7].

Neuronal protection is a term that conglomerates a variety of strategies focused on reducing cell death after an ischemic event without affecting tissue reperfusion. To date, several compounds have been proposed to block the pathway leading to ischemia-induced cell death at different steps of the ischemic cascade. These interventions range from physical approaches such as hypothermia to the use of pharmacological drugs [8]. Despite the positive results obtained in preclinical studies, there is no protective compound for stroke patients yet, due to the narrow therapeutic window, ability of the drug to reach brain targets, side effects, or short half-life after drug administration [8]. 

Neurorepair strategies, usually based on cell therapies or growth factor administration, involve the restoration of brain function, either by regeneration of the damaged cerebral tissue or by the establishment of alternative neural pathways or synapses (referred to as brain plasticity). The therapeutic window for these therapies is wider than the one in thrombolytic or neuroprotective approaches. The aim of the treatments for neurological function recovery after stroke is not restricted to the neurons; it is more focused on the neurovascular unit, including procedures that enhance synaptogenesis and angiogenesis. Thus, neurorepair treatments may use stem cells and pro-neurogenic, pro-angiogenic, and/or pro-synaptogenic drug delivery, among others [9].

### 1.2. Nanomedicine

Nanotechnology emerged in 1959 as a new area of study that involved the creation of materials or systems at the nanometer scale, defined as nanoparticles (NPs). Later, this technology was applied to the medical field, opening a new era in the diagnosis and treatment of many diseases, currently known as nanomedicine [10]. The development of NPs for clinical use has allowed, for instance, the encapsulation of hydrophilic drugs, a decrease in the therapeutic doses, a reduction in side effects, an improvement in the biocompatibility and therapeutic effect, or targeting and delivery of the drug to the desired region [11].

Over the last few years, the complexity of nanosystems has increased exponentially. Initially, the first formulations were mainly focused on the encapsulation of drugs for increasing their safety and efficacy; however, the new advances in the field of nanomedicine have allowed the development of “smart” delivery nanoplatforms that, after administration, can specifically recognize the pathological region and induce a controlled drug delivery that can be induced by internal or external stimuli. Internal stimuli involve chemical and biochemical changes in the targeted region, such as pH, temperature, redox, and ionic changes, or even enzymatic activities, whereas external stimuli comprise, for instance, light, magnetic field, or ultrasound [11]. 

Owing to the versatility of nanomedicine, as observed in other pathologies, this technology has been widely applied in the field of stroke, not only for diagnosis but also to improve the efficacy of thrombolytic therapy and protective drugs, as well as to help in the development of recovery therapies.

## 2. Nanomedicine for Stroke Diagnosis

Computed tomography (CT) and magnetic resonance imaging (MRI) have revolutionized ischemic stroke diagnosis and management. Initially restricted to structural imaging to exclude bleeding, these imaging modalities can now detect intracranial vessel occlusion, evaluate the ischemic penumbra to select candidates for thrombectomy, and play a key role in identifying stroke etiology. Molecular imaging has the potential to further expand the information provided by CT and MRI by revealing the biological processes that constitute potential diagnostic or therapeutic targets [12]. The use of nanotechnology to design contrast agents has led to significant advances in the field [13]. 

The recent development of a new family of contrast agents for MRI based on micro-sized particles of iron oxide (MPIOs) has allowed a large increase in the sensitivity and specificity of this imaging modality, paving the way for clinical application [14]. The use of larger particles with a diameter of at least 1 µm has several advantages compared to the more classically used ultrasmall superparamagnetic iron oxide particles (20–50 nm). Indeed, it prevents the passive leakage of particles in the brain parenchyma and increases the payload of the contrast material per particle [15]. Using MPIOs coupled with monoclonal antibodies, non-invasive detection of specific proteins expressed by the cerebrovasculature has been demonstrated in neurovascular disorders in the last few years [16]. 

The combination of in vivo MRI with NP contrast agents has allowed the clarification of many aspects of post-stroke inflammation, which is one of the main pathogenic processes occurring in the acute and subacute phases of stroke [17]. Monitoring the temporo-spatial regulation of the inflammatory reaction in the affected brain parenchyma could have significant clinical applications, such as identifying patient subsets that could benefit from immunomodulatory treatments. In this regard, the endothelial cells of the blood–brain barrier (BBB) are key players in post-stroke inflammation by mediating the diapedesis of leukocytes from the blood to the brain through the expression of adhesion molecules. Since these adhesion molecules are easily accessible by large contrast-carrying particles, they constitute interesting targets for molecular imaging. Using MPIOs coupled with monoclonal antibodies targeting vascular cell adhesion molecule-1 (VCAM-1), molecular MRI has provided evidence for the existence of a new concept defined as “inflammatory penumbra” in ischemic stroke [18]. In mice, 24 h after ischemic stroke, this technique unveiled an inflammatory area at risk, surrounding the initial ischemic lesion, which was secondarily infiltrated by lymphocytes and ultimately recruited by the ischemic core [18]. By analogy with the ischemic penumbra, the mismatch between the VCAM-1-overexpressing region (revealed by molecular MRI) and the ischemic core (revealed by diffusion-weighted imaging 24 h post onset) was called the “inflammatory penumbra”. Interestingly, the size of this “inflammatory penumbra” varied according to the duration and subtypes of ischemic stroke in experimental models, suggesting that there could be a wide variability in the intensity and spatial extent of the inflammatory reaction between patients. 

Besides VCAM-1, other adhesion molecules are involved in the diapedesis of leukocytes from the blood to the brain. For instance, Deddens and co-workers performed molecular MRI of intercellular adhesion molecule-1 (ICAM-1) in an experimental model of ischemic stroke in mice. They used MPIOs targeting ICAM-1 to reveal the overexpression of ICAM-1 by activated endothelial cells at different time points after ischemic onset [19]. They found that ICAM-1 expression was maximal at 48 h and extended in the peri-infarct area, in line with the “inflammatory penumbra” concept. Whether VCAM-1 and ICAM-1 imaging methods provide differential information on stroke, pathophysiology remains to be explored. Moreover, Quenault et al. demonstrated that molecular MRI of P-selectin using MPIOs could be employed to diagnose a transient ischemic attack (TIA). In a TIA, the duration of ischemia is too short to induce changes on an unenhanced MRI scan. Interestingly, experimental studies have revealed that P-selectin is overexpressed for at least 24 h in the affected vascular territory. Using molecular MRI of P-selectin, it is therefore possible to detect the endothelial activation triggered by the TIA and distinguish TIA from stroke mimics, such as epilepsy or migraine [20]. 

Molecular MRI can also aid in the etiologic assessment of ischemic stroke. Indeed, it has been previously demonstrated that ruptured atherosclerotic plaques overexpress endothelial activation markers. Using MPIOs targeting P-selectin and VCAM-1, McAteer et al. demonstrated that it is possible to detect the activated endothelium inside atherosclerotic plaques using MRI [21]. Molecular MRI of endothelial activation in stroke patients could thus be utilized to identify the culprit vascular lesion and therefore better characterize stroke etiology.

Even if its sensitivity to detect contrast material is lower than that of MRI, CT can also be used for molecular imaging using NPs. This has been demonstrated in experimental models of carotid artery thrombosis and embolic ischemic stroke [22]. Kim et al. showed that fibrin-targeted gold NPs can reveal cerebral thrombus as a high-density endovascular material on CT images. In acute stroke settings, this would allow the assessment of thrombus burden and monitoring of thrombolytic therapy in a non-invasive manner.

Given the demonstration of the high sensitivity and specificity of molecular imaging of inflammation offered by MPIO-enhanced molecular MRI in experimental models, efforts are ongoing to translate this method to clinical imaging. Indeed, the MPIOs used in preclinical studies are non-biodegradable because of their coating and inner structure. In this context, the development of biocompatible MPIOs is mandatory. To overcome this limitation, Perez-Balderas et al. recently reported the production of multimeric magnetite particles that form large MPIO-like particles that are biodegradable [23]. In an experimental model of neuroinflammation, they demonstrated that this method allowed non-invasive imaging of activated endothelial cells.

## 3. Nanoparticles for Recanalization Therapies

Approximately 85% of all stroke cases are ischemic strokes that occur due to the obstruction by blood clots within a blood–brain vessel. To date, rtPA remains the only thrombolytic drug for the treatment of acute ischemic stroke [24]. rtPA is a fibrin-specific serine protease that activates the endogenous proenzyme plasminogen and converts it to the active form plasmin, thus degrading the thrombus fibrin network. In addition to the risk of hemorrhagic transformation (HT), systemic delivery of rtPA has to be administered intravenously, 10% as bolus, and 90% as infusion due to its short half-life (3.5 min, with a clearance of 75% in the first 8 min). This short half-life is caused by the efficient inactivation of resident blood enzymes, such as plasminogen activator inhibitors (PAI-1 and PAI-2) [25]. Administration of rtPA is also related to neurotoxic side effects due to the activation of microglia, N-methyl-d-aspartate (NMDA) receptors, and metalloproteinases [26].

Owing to the risk of HT, rtPA infusion is restricted to a narrow therapeutic window (4.5 h of stroke symptom onset when injected alone and 6 h when combined with mechanical thrombectomy). Unfortunately, only approximately 30% of patients treated with rtPA achieve full or partial recanalization after intravenous administration. In addition, rtPA administration has to be done by experienced neurologists after exclusion of intracranial hemorrhage by CT or MRI, which increases the “door to needle time.” All these limitations mean that less than 10% of stroke patients can benefit from this treatment [27,28]. 

In the past two decades, studies have focused on extending the rtPA therapeutic time window beyond 4.5 h or exploring alternative thrombolytic agents, while most drugs have failed in clinical trials [29]. Currently, endovascular recanalization therapy (ERT) by mechanical thrombectomy has emerged as a new treatment for acute ischemic stroke. ERT has the advantage of a higher recanalization rate for proximal intracranial artery occlusions that are usually resistant to intravenous rtPA. However, despite the clear benefits of ERT, this novel recanalization strategy still has important limitations. For instance, it is not effective when the occlusion is in the distal cerebral arteries (small arteries), which occurs in more than 30% of ischemic strokes. The use of ERT may also release microclots with the risk of subsequent distal strokes; therefore, it is usually employed in combination with rtPA to improve the rate of reperfusion as well as to dissolve the microclots produced during mechanical thrombectomy. In addition, ERT requires the availability of personnel for imaging, surgery, and endovascular facilities, which limits its use to the main cities with large hospitals [30]. 

This shows that rtPA perfusion remains the gold standard for acute ischemic stroke and is a well-established procedure widely used in all hospitals [31].

The last few years have seen a new clot-busting drug, named tenecteplase, that is already accepted in the treatment of heart attack. Based on its chemical properties, it could be a safe and effective drug for treating stroke, becoming a serious rtPA competitor.

**Figure 4:**
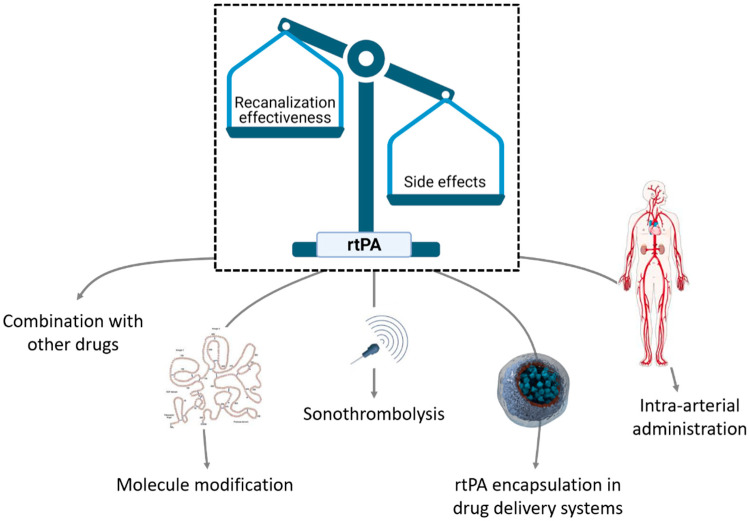
To increase the recanalization effectiveness of recombinant tissue plasminogen activator (rtPA) and decrease the side effects, some approaches have been carried out. For example, combination with other drugs, the chemical customization of the molecule, intra-arterial administration, combination with sonothrombolysis, and the encapsulation of the drug in delivery systems. Figure created with BioRender.com.

In addition to the new advances in the chemical customization of rtPA, nanomedical approaches for the targeted delivery of thrombolytic agents have been intensively investigated. These nanomedical approaches are focused on targeting the drug to the arterial clot region to increase the thrombolytic efficacy and induce controlled drug release to reduce the risk of HT associated with delayed administration [32] (Figure 4).

### 3.1. Nanocarriers for rtPA

**Figure 5:**
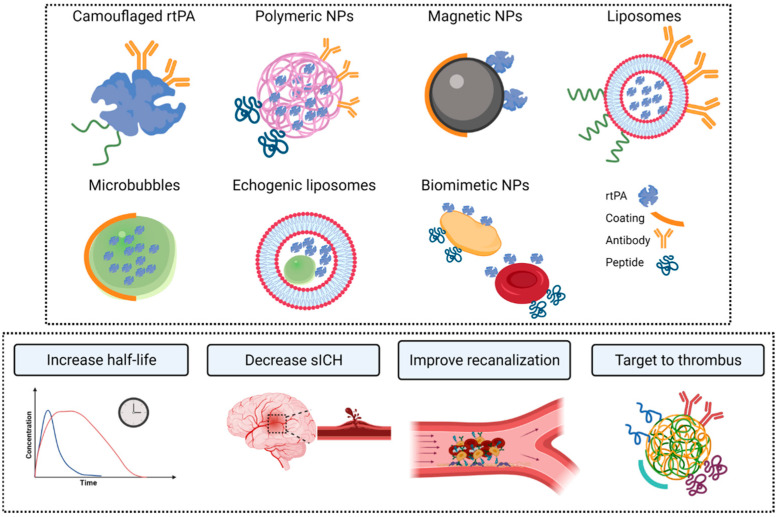
Examples of nanodelivery systems for rtPA to extend the half-life of the therapeutic agent, to reduce secondary effects, improve the recanalization, target the therapy, and improve the efficacy. Figure created with BioRender.com.

To date, many nanomedical carriers have been developed to encapsulate and optimize the efficacy and safety of rtPA, such as liposomes, polymeric NPs, magnetic NPs (MNPs), microbubbles, and echogenic liposomes, with different levels of success [32,33] (Figure 5).

Liposomes, comprising an amphiphilic phospholipid bilayer and a hydrophilic aqueous core, can encapsulate both water-soluble and water-insoluble compounds. Liposomes were one of the first nanosystems employed as drug delivery systems for rtPA. Heeremans et al. showed, for example, that rtPA-loaded liposomes have a better thrombolytic effect than free rtPA [34]. In comparison with other nanocarriers, liposomes also have many advantages such as biodegradability, biocompatibility, low immunogenicity, and flexibility in coupling with site-specific ligands. 

Both synthetic and natural polymers have been used to fabricate NPs for thrombolytic therapy because of their biocompatibility and biodegradability. Synthetic polymers can be easily functionalized, and the resulting NPs can be customized in terms of size, porosity, and hydrophobicity. Poly(lactic-*co*-glycolic acid) (PLGA), an FDA-approved polymer, represents one of the most common polymers used for rtPA delivery. Natural polymers such as chitosan and gelatin have also been investigated as alternative materials for rtPA delivery [33]. Although these natural polymers are biocompatible and biodegradable, they have variable compositions depending on the natural source and they need to be standardized. 

Conjugation of rtPA with MNPs has been frequently employed in thrombolytic therapy. This strategy allows the evaluation of the fate of the treatment based on the capability of the particles to act as a magnetic resonance contrast agent. In addition, under the application of a local magnetic field, the thrombolytic effect of the drugs tends to accumulate at a specific site, which is a favorable property for targeted thrombolysis [35]. Currently, iron oxide NPs are the most explored MNPs because of their biodegradability and known metabolic pathways. Among iron oxides, magnetite (Fe_3_O_4_) and maghemite (γ-Fe_2_O_3_) are very popular candidates that possess suitable magnetic properties for biomedical applications [33].

Microbubbles (MBs) are microspheres filled with gas or air used as contrast agents for eco-Doppler imaging owing to their acoustic characteristics. The combination with rtPA to evaluate reperfusion success showed that these MBs were able to accelerate the thrombolytic activity, which was attributed to stable and inertial cavitation, resulting in microstreaming and erosion of the clot surface, which enhanced the penetration of thrombolytic agents into the clots. These results led to the fabrication of MBs loaded with rtPA to optimize the thrombolytic efficacy and potentially reduce the hemorrhagic risk under ultrasound exposure [36,37].

Similar to MBs, echogenic liposomes are multifunctional phospholipid bilayer-encapsulated vesicles, which can be used as contrast agents for sonography and drug delivery, including rtPA. The exposure of rtPA-loaded echogenic liposomes to ultrasound can disrupt the lipid shell and hence trigger drug release. As a result, under ultrasound stimuli, rtPA can be released locally, thus increasing the concentration of rtPA in the area of thrombus, reducing the required therapeutic dose of rtPA and consequently the risk of hemorrhage. Moreover, the gas encapsulated in echogenic liposomes can exert a cavitation-related mechanism, leading to enhanced thrombolytic effects [38].

### 3.2. Clot Targets for rtPA Nanocarrier Vectorization

**Figure 6:**
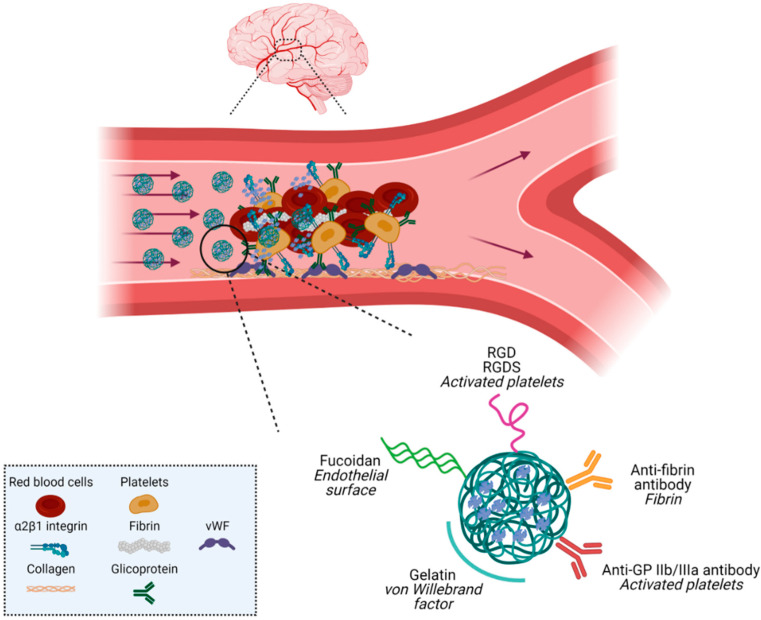
Strategies to target the nanosystems to the thrombus. Figure created with BioRender.com.

Although passive targeting of rtPA nanosystems has shown promising thrombolytic efficacy, recent actively targeted nanocarriers have been designed. Active targeting permits drug accumulation specifically at the thrombus site and has the potential to enhance enzyme penetration into deeply localized thrombi. Many of the targets are related to clot components, such as activated platelet receptors, thrombin, fibrin, red blood cells, or von Willebrand factor (vWF) [32] (Figure 6).

After thrombus formation, thrombin and collagen included in the clot cause the activation of the platelet glycoprotein (GP) IIb/IIIa receptor, which leads to changes in the platelet cells from an inactive to an active state. The critical role of these receptors in thrombus formation has provided an excellent target for nanosystems designed for thrombolytic therapy. For example, the peptides arginine–glycine–aspartic acid (RGD) or arginine–glycine–aspartic acid–serine have been used to target NPs to the GP IIb/IIIa receptors of the clot region. Thus, decorating the liposome surface with RGD peptide resulted in an increase in fibrinolytic activity and targeting of platelets [39]. Similar results on clot dissolution were also observed in other independent studies employing PLGA and chitosan NPs customized with the RGD peptide [40]. These peptide sequences have been used to target magnetic NPs with rtPA, allowing the monitoring of thrombolysis by MRI [41]. P-selectin, a transmembrane glycoprotein of activated platelets, is another clot target for rtPA vectorization using the polymer fucoidan [42].

Thrombin is other an abundant component of the clot that has been widely used to target thrombolytic NPs, as shown by Absar et al. [43].

Fibrin (also called Factor Ia), which is involved in blood clotting, is formed by the action of the protease thrombin on fibrinogen, which causes it to polymerize. The polymerized fibrin, together with platelets, forms a hemostatic plug or clot over a wound site. Fibrin appears only in the clot region, but is not present in blood circulation and in normal tissue, which makes it a suitable goal for fibrinolytic nanosystems [43]. Customization of thrombolytic NPs with antibodies against different fragments of the fibrin, such as B-chain or D-dimer, confirmed the higher efficacy and safety of these formulations with respect to free rtPA administration [44,45]. Recent studies reported by Huai-An et al. described that the association between magnetic NPs coated with PLGA, rtPA, and a peptide against the fibrin clot region was linked with higher thrombolysis rate in vitro and reduction of clot lysis time in vivo [46].

Gelatin nanostructures were also designed to encapsulate rtPA for controlled drug delivery. Gelatin is used because of its biocompatibility, and its ability to bind to the vWF, an endothelium factor that participates actively in clot formation. Uesugi and coworkers used this polymer to encapsulate rtPA and generate ultrasound-responsive NPs. These assays showed that rtPA activity was suppressed around 50% in these gelatin complexes, and the activity was completely recovered on application of ultrasound (2 MHz, 0.72 W/cm^2^). In vivo analysis in swine confirmed the tendency of NPs to bind to the clots, and increase the recanalization effectiveness under ultrasound radiation [47,48].

The interest in biomimetic NPs to encapsulate rtPA has increased significantly in recent years. Biomimetic NPs, mainly based on the use of cell membranes of both platelets and red blood cells, are invisible to the phagocytic system, and aim at increasing the half-life of the NPs and improving the targeting efficacy to the thrombus region. Promising results were obtained for the fibrinolytic activity with rtPA conjugated on the surface of red blood cell membranes [49]. One of the first successful studies using platelets to encapsulate rtPA was carried out by Hu et al., who published a biomimetic NP based on platelet membrane coating. This novel design showed an improvement in the efficacy and safety of the thrombolytic activity of rtPA in stroke models. The efficacy of this “smart” strategy was also enhanced by the affinity binding of the platelet membrane to the clot components [50].

### 3.3. Triggering Controlled Release

The controlled release of an encapsulated drug (such as rtPA), has been one of the most important advantages in the design of new nanosystems. This drug-controlled release allows for focus on the effect of the drug on a specific target, increases the efficacy of the drug, and importantly, reduces the side effects. 

**Figure 7:**
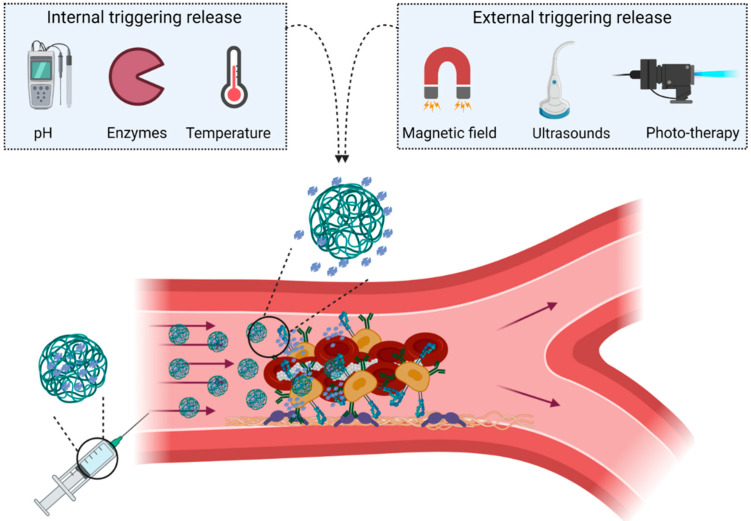
Stimuli-responsive nanosystems. The release of the nanosystems in the desired area can be triggered by internal and/or external stimuli. Enzymes, pH, and temperature are the most common examples of internal stimuli. Externally, the release can be triggered by magnetic fields, ultrasound, or photo-therapy. Figure created with BioRender.com.

Two types of stimuli are used to induce the release of the encapsulated drug: internal and external stimuli (Figure 7).

Molecular changes related to thrombus formation, metabolic events of the ischemic cascade, biochemical changes in the microenvironment, or shear stress are some examples of internal stimuli methods that can be found in the literature. 

Thus, thrombus formation is associated with increased levels of oxidative species, such as hydrogen peroxide (H_2_O_2_), which also lead to platelet activation and recruitment. Demonstration of H_2_O_2_ as a trigger for thrombolysis was performed by the Jung and Kang groups [51,52], who fabricated nanostructures capable of releasing thrombolytic treatment content in the presence of H_2_O_2_, and with the advantage of decreasing the H_2_O_2_ levels in the surrounding clot medium, obtaining antioxidant and anti-inflammatory effects for thrombolysis.

The acidification of the occluded region is another metabolic process related to arterial thrombus formation, and the pH difference between the region of the thrombus and normal tissues could be used as an endogenous stimulus. Some cases of this method have been reported by Wei et al., who designed pH-responsive nanosystems able to protect drug inactivation in blood, increase the thrombolysis efficacy, and reduce the risk of hemorrhage in a model of thrombosis [53].

Making use of the increased number of different enzymes in the clot region has been an interesting strategy to induce the delivery of encapsulated drugs. Gunawan et al. developed polymeric capsules that specifically target the GP IIb/IIIa receptor and are degraded by the serine protease thrombin, leading to the release of thrombolytic treatment loaded into the nanosystem only around the thrombus [43]. Another blood-clotting enzyme that has been successfully used to destabilize microparticles and release the thrombolytic agent is phospholipase-A2 [54]. Metalloproteinases are other critical enzymes that increase in the ischemic region related to the degradation of the extracellular matrix of the BBB and are associated with poor outcome in stroke patients. The protease activity of this enzyme has been used to destabilize and trigger the release of encapsulated drugs from NPs of a collagenous nature [55,56].

The occluded region is widely related to the shear stress caused by the reduction of the vessel lumen. Thus, NPs that respond to fluidic changes have been used as “smart” options to induce drug release in this zone. For instance, Korin and colleagues fabricated microaggregates of NPs with rtPA on the surface, which broke up and released rtPA on exposure to high shear stress in the occluded vessels, lowering the therapeutic dose of the drug required to induce the thrombolytic effect [57].

As previously cited, alternative strategies to spatially and temporally control the delivery of encapsulated thrombolytic agents are the use of external stimuli, which involve physical processes such as light, temperature, magnetic fields, or ultrasounds.

Photo-therapy is a non-invasive strategy in which agents can convert the irradiation with a local temperature increase. For example, l-tetradecanol has been used to fabricate near-infrared-responsive nanocarriers composed of gold mesoporous silica nanospheres for the encapsulation of thrombolytic drugs. This component changes its state when it reaches 38 °C, allowing the release of the thrombolytic treatment when it is irradiated, resulting in an increase in the arterial recanalization rate in the in vivo model, and less severe side effects in terms of bleeding [58]. In 2019, Zhang et al. published a report on RGD-modified mesoporous carbon nanospheres that could carry out thrombolysis by hyperthermia under near-infrared irradiation. The nanospheres were administered in a thrombosis model in mice, showing an accumulation in the thrombus, which was disaggregated due to the local increase in temperature caused by the irradiation [59].

There are also some thermo-sensitive polymers that change their physico-chemical properties depending on the temperature, which can be used to create temperature-responsive NPs. For instance, thermosensitive liposomes have been developed to achieve on-demand rtPA release and site-specific targeted thrombolysis [60].

Another external strategy to induce controlled drug release in the clot region consists in the application of a magnetic field using magnetic Fe_3_O_4_ NPs with rtPA [61]. In recent years, MNPs have not only been used to carry the treatment but also to create a local hyperthermia that accelerates thrombolysis [62]. Fe_3_O_4_ rods are elongated NPs that have a larger and stronger contact area with the endothelium. These rod NPs were tested in vivo, where a magnetic field was used to guide the occluded vessel, and the clots were dissolved by rotation of the rods in combination with rtPA [63]. 

Lastly, one of the most interesting options with potential clinical applications is ultrasound-mediated release, which is a non-invasive and safe method that is also used for eco-Doppler clinical imaging. The combination of ultrasound application and free rtPA administration, clinically defined as sonothrombolysis, has been observed to increase the thrombolytic effect due to thermal, mechanical, and cavitation effects. These studies motivated scientists to encapsulate rtPA in microbubbles or echogenic liposomes, and showed that ultrasound external radiation facilitates the increase in thrombolysis, compared with rtPA administration alone, in both in vitro and in vivo stroke models [64]. 

**Figure 8:**
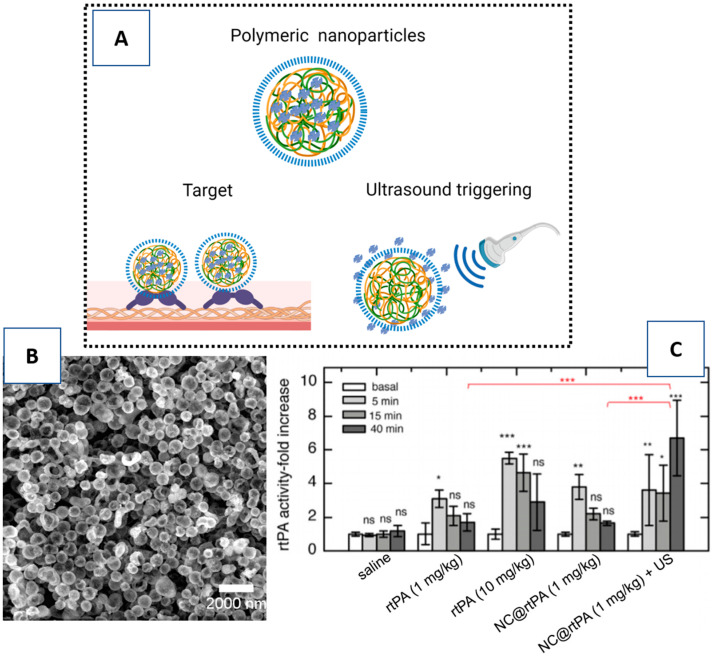
Echogenic polymeric nanocapsules covered by gelatin and encapsulating rtPA. (**A**) A schematic representation of echogenic polymeric nanocapsules covered by gelatin and encapsulating rtPA. The gelatin binds to the von Willebrand factor of the thrombus, and ultrasound triggers the release of rtPA. (**B**) Scanning electron microscopy (SEM) image of the nanocapsules. (**C**) In vivo release of rtPA from nanocapsules by ultrasound (US). Ns (no significance); * *p* < 0.05; ** *p* < 0.01; *** *p* < 0.001. Figure (**A**) created with BioRender.com. Figure (**B**,**C**) modified from [65].

Recent studies have confirmed the utility of the layer-by-layer technique to encapsulate rtPA in microcapsules in a way that does not interfere with the thrombolytic activity of rtPA and that ultrasound is capable of inducing the controlled release of rtPA. The main advantage of these encapsulation systems is that they allow the encapsulation of any drug (hydrophilic or hydrophobic), ranging from small molecular weight (MW) molecules (for example, dyes) to large MW molecules, such as proteins, enzymes, and oligonucleotides, and may incorporate inorganic NPs into their shell in order to provide, for instance, MRI contrast by MNPs [65] (Figure 8).

## 4. Nanoparticles as a Therapy for Ischemic Brain Protection

Ischemic neuroprotection involves searches to find novel therapies that have the potential to preserve brain tissue and improve the overall outcome in stroke patients. Initial studies have focused on neuronal death as the main cause of neuronal deficits in stroke patients; however, in recent years, the focus has widened to include other neural cells such as astrocytes, pericytes, and endothelial cells, which together form the neurovascular unit.

**Figure 9:**
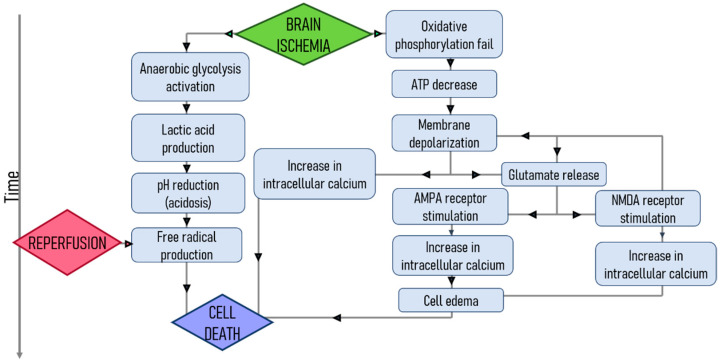
Major events that occur after cerebral ischemia, leading to neuronal death. Figure created with BioRender.com.

In contrast to reperfusion therapies, in which strategies have a specific goal of recanalization of the occluded artery, neuroprotection is composed of a pool of approaches that focus on different sub-processes involved in the ischemic cascade, such as inflammation, oxidative stress, endothelial damage, or excitotoxicity [8] (Figure 9).

In-depth studies of the ischemic cascade have allowed us to understand in detail many of the molecular and cellular events that lead to the death of the neuronal tissue and the consequent neuronal deterioration of the patient. Based on this knowledge, hundreds of neuroprotectant drugs have been developed with successful results in both in vitro and in vivo analysis; however, to date, none of these agents has shown beneficial effects in clinical trials despite compelling preclinical evidence [8]. Many reasons have been suggested to explain this translational gap, such as the accuracy of the experimental models, quality of study design, or the selection of the outcome assessments [66]; however, limitations such as neurotoxic side effects, BBB permeability issues, and the short therapeutic time window of the pathology have significantly exacerbated the failure of these protective therapies.

Advances in research on neuroprotection for stroke have shown that nanomedicine acts as a versatile approach to solve some of the limitations cited for protective drugs [67]. As has been described for thrombolytic drugs, NPs allow drug encapsulation by reducing the therapeutic dose and side effects, solving the issue of biocompatibility, and changing the composition, size, or surface coating to allow different strategies to bypass the BBB to reach the neuronal target region [67]. However, NP composition and design must be carefully selected for the specific treatment and purpose. For instance, biodegradable polymers have been developed as potential carriers for drug delivery to the central nervous system because of their high biocompatibility, low toxicity, and good sustained-release profiles. Other approaches, such as exosomes or cell biomimetic NPs, provide targeting advantages, reduce immune system trapping, and increase circulation time [11,67]. 

Below, we summarize some neuroprotective approaches through the use of NPs according to the ischemic molecular target.

### 4.1. Inflammation

Inflammation is a critical and multifactorial process related to stroke outcomes with both deleterious and beneficial effects, depending on the temporal evolution of the ischemic injury [68,69,70]. In addition, cerebral ischemia causes local inflammation involving microglia, neurons, and astrocytes, and systemic leukocyte infiltration promoted by brain chemotaxis [69]. In this regard, nanomedicine, based on anti-inflammatory therapy, has received special attention for the development of targeting strategies to block inflammatory processes directly in the brain or in the systemic immune system [70].

Membrane biomimetic NPs such as exosomes (biocompatible lipophilic NPs from mesenchymal stromal cells) have been used as excellent nanocarriers for the transportation of anti-inflammatory drugs to ischemic regions because of their lipidic properties that cross the BBB. These lipidic particles were employed to deliver inhibitors of proinflammatory factors, such as NF-κB, in brain ischemic injury [71]. Another example of a biomimetic nanocarrier is based on the ability of neutrophils to cross the inflamed endothelium and the BBB to the brain parenchyma. Feng et al. designed a neutrophil-like cell membrane-coated NP for the transportation of protective nanozymes to the brain, with potential applications for stroke treatment [72].

At the systemic level, the vascular endothelium has been widely explored as a specific target for the design of delivery platforms, mainly related to inflammatory pathologies [73,74]. The vascular endothelium plays an essential role in the regulation of inflammatory processes, as in the cases of myocardial infarction and stroke. In response to local pro-inflammatory stimuli, the endothelial expression of cell adhesion molecules is upregulated to mediate interactions with immune cells [75]. This allows for leukocyte adhesion to the endothelium, followed by extravasation through the endothelial cell layer to the site of inflammation [75]. Various vascular inflammatory proteins have been described that participate in this response. Two heavily investigated receptors are ICAM-1 and VCAM-1 [76]. The specific upregulation of these two inflammatory-mediated vascular proteins has been used to target not only nanodelivery drug systems [77] but also for the in vivo visualization of biological processes [18] (and summarized in previous sections). 

**Figure 10:**
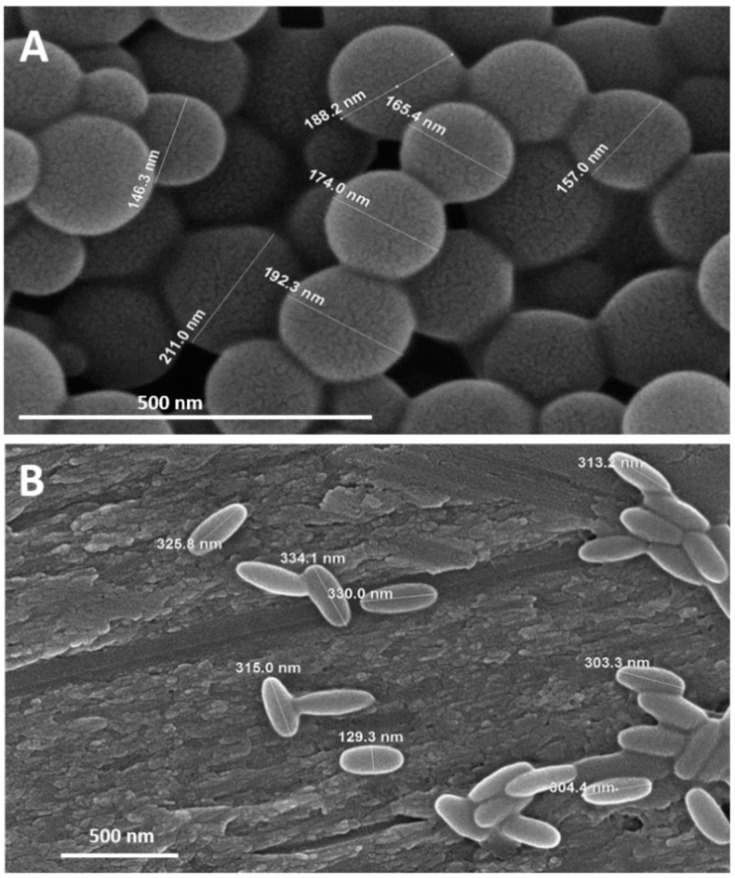
Scanning electron micrographs of spherical (**A**) and rod-shaped nanoparticles (**B**). Reproduced with permission from [81], Elsevier, 2019.

Multiple types of nanosystems for immune-protective drug delivery have been described using selective endothelial cell receptors as targets; however, one important physical parameter that can affect the success of NPs in reaching their target in the vascular endothelium is the blood flow rate and vessel tortuosity. Several aspects can be modified while designing an NP that can improve the binding rate after in vivo administration, such as size, shape, and charge. The ideal combination of these factors can determine the success of binding to a cellular target [78]. The overall particle geometry is a key parameter for endothelial targeting [79] when comparing the classical spherical shape with rod-shaped particles [80]. Indeed, da Silva-Candal et al. demonstrated that the antibody surface density of rod particles with anti-VCAM-1 coating is higher than that of the spherical shape, enhancing the in vivo targeting in the inflamed endothelial brain [81] (Figure 10). 

Shape is also a critical aspect that effects to the biodistribution of the NPs. Thus, after administration, NPs are rapidly sequestered by immune cells such as macrophages, reducing their biodistribution and the circulation half-life. The rate of internalization in the macrophage cells is closely related to the particle geometry and the contact angle, in a way that spherical particles internalize substantially faster than asymmetric-shaped particles. It could be assumed the rod geometry as the optimal design; unfortunately, considering the therapeutic potential of NPs, the spheres have a superior drug-loading capacity than non-spherical shapes [82].

These results suggest that an appropriate selection of the NP shape is critical for designing drug delivery nanosystems or contrast agents related to neurovascular inflammation processes such as stroke [81].

### 4.2. Oxidative Stress

Tissue and cell damage after ischemic stroke and reperfusion leads to the production of reactive oxygen species (ROS), activating apoptosis, necrosis, and autophagy pathways that worsen the injury. In this regard, nanomedicine has contributed substantially to this field by developing NPs with antioxidant treatment cargos [83,84].

An antioxidant is defined as any substance or compound that, at a low concentration, is able to inhibit the oxidation of suitable substrates. The antioxidant is a stable molecule, which donates an electron to unwanted free radical species, leading to their neutralization, and blocks or reduces the risk of oxidative damage to biological systems. Ascorbic acid, melatonin, and many others are biological molecules synthesized by the cells for self-protection against ROS, but the list of natural or synthetic antioxidant agents is much longer when NPs are involved. The combination of antioxidant agents and nanotechnology has been explored in stroke by using compounds such as resveratrol, with successful protective results in in vitro and in vivo stroke models [83,85]. Some NPs also have the capability to act as ROS scavengers, mimicking the therapeutic effect of an antioxidant molecule, owing to their intrinsic physicochemical properties. An excellent example is cerium oxide NPs or nanoceria, a material that has been used extensively owing to its biocompatibility and catalytic antioxidant properties [86].

### 4.3. Glutamate Excitotoxicity

Glutamate excitotoxicity is a primary contributor to ischemic neuronal death following a stroke event. Several strategies have been developed against it; however, none of them has shown positive results in clinical practice to date [87]. The major receptor involved in glutamate-mediated neuronal damage is NMDAr, an important receptor essential for neuronal activity and plasticity (namely, learning and memory formation). Studies have shown that increased activation of NMDAr by high levels of glutamate plays a significant role in neuronal excitotoxicity by a receptor-mediated influx of calcium, which leads to the downstream activation of other mechanisms, including ROS production, mitochondrial toxicity, and other subsequent pathways of the ischemic cascade [87]. 

The inhibition of glutamate excitotoxicity and NMDAr overactivation has been an important challenge for nanomedicine focused on the blockade of the ischemic cascade. Inhibition of NMDAr is a complex pharmacological process, as this receptor is essential for proper neuronal activity, which has led to the development of nanocarriers loaded with NMDAr inhibitors that selectively target the affected ischemic region. In line with this, a smart strategy was developed by Savchenko et al., who designed a hybrid nanodrug composed of memantine (an effective inhibitor of NMDAr) linked to a gold NP. The new formulation described by these authors caused selective inhibition of extrasynaptic NMDAr activation, while having no effect on physiological synaptic transmission [88].

### 4.4. Combination of Thrombolytics and Neuroprotectants in Nanomedicine

The side effects related to rtPA administration, such as neurotoxic effects, ROS formation, inflammation, or BBB damage [89], have stimulated a design of new nano-formulations based on the combination of thrombolytic treatment and neuroprotectants. Fukuta et al. studied the co-administration of liposomes with an effective neuroprotectant, fasudil, and rtPA, prolonging the therapeutic window due to a decrease in the rtPA side effects [90]. Moreover, the encapsulation of rtPA in self-assembled antioxidant NPs was synthesized and tested in a rodent stroke model with significant infarct volume reduction and neurological deficit improvement [91]. In a recent experimental study, a new bioengineered nanoplatelet was developed for sequential site-specific delivery of rtPA and neuroprotectant ZL006e, resulting in a decreased ischemic volume and ROS levels in a rat model of brain ischemia [50].

## 5. Nanomedicine for Stroke Recovery Therapies

Stem cell-based therapies have emerged as a promising tool for recovery treatment in delayed phases of stroke owing to their multipotentiality, ability to release growth factors, and immunomodulatory capacities. Thus, this transdifferentiation can produce cells with a neural lineage, induce neurogenesis, angiogenesis, synaptogenesis, and activate endogenous restorative processes through the production of different cytokines and trophic factors. Moreover, the regulation of CBF, the BBB, and other neuroprotective mechanisms, such as the reduction of apoptosis, inflammation, demyelination, and the increase in astrocyte survival have been shown to be beneficial mechanisms after stroke [92]. 

Despite special attention to stem cells as a promising therapeutic candidate for stroke, parameters such as administration route or cell dosage are still under discussion. MRI in combination with contrast agents or NPs for in vivo cell tracking is widely used to evaluate these parameters [3,93].

### 5.1. MRI Contrast Cell Agents

MRI contrast agents for cell tagging require NPs with special characteristics, such as high magnetization values and small and narrow size distributions. Thermal decomposition and chemical coprecipitation are the most widely used procedures. The coprecipitation technique is probably the simplest and most efficient chemical route to obtain magnetic NPs. The main advantage of this method is that a large number of particles can be synthesized easily and economically [94]. However, aside from the magnetic properties, the targeting surface coating of the magnetic particles must be non-toxic and biocompatible to be useful for localized delivery [95]. 

Among all the coating possibilities, dextran is one of the most widely used polymers for MNP coating because of its biocompatibility. This polysaccharide polymer is composed exclusively of α-d-glucopyranosyl units with varying degrees of chain length and branching. In 1982, Molday and Mackenzie were the first to report the formation of magnetite in the presence of dextran 40,000 [96]. The synthesis of dextran-coated NPs is an in situ procedure, and the effect of reducing the terminal glucose of dextran on the formation and stability of dextran-coated superparamagnetic NPs has been demonstrated to be significant for particle size, coating stability, and magnetic properties [94].

For cell labeling applications, in addition to the selection of optimal material and biocompatible coating, transfection agents (TAs) or electroporation processes are required to allow NPs to enter the cells, which is a critical step [97,98]. For instance, Tas, such as poly-l-lysine (PLL) are toxic to cells; therefore, in experimental studies based on cell tracking, it is necessary to determine the appropriate ratio of NPs/TAs to efficiently label cells and minimize toxic side effects. In addition, stem cells are sensitive to culture medium and can exhibit different behaviors when their composition is changed; therefore, cellular phenotype and functionality (for example, growth factor release) after labeling must be verified to guarantee the biocompatible nature of the material [97]. Other parameters that are important to properly characterize the cell labeling are the amount of NP uptake, ROS generation, and intracellular NP localization [99].

Other methods to synthesize coated magnetic NP cell labeling are much more complex and require resolving of solubility issues. For instance, Jain et al. [100] developed a novel oleic acid (OA)–Pluronic-stabilized iron oxide MNP formulation, in which the OA shell surrounds the iron oxide NPs, and then the polymer (Pluronic) is anchored at the interface of the OA shell to confer an aqueous dispersity to the formulation. Pluronic coating also has the advantage of not requiring transfection agents or electroporation for cell labeling [101].

### 5.2. MNPs for MRI Cell Tracking in Stroke 

Relatively few studies have compared the different possible routes of stem cell administration for stroke recovery. The first studies that used stem cells for cerebrovascular diseases involved looking for a neuronal replacement, so an intraparenchymal injection was chosen as the most direct route for cell engraftment. These studies showed that stem cells not only survived but migrated to the affected zone [102,103]. However, this choice is not the most suitable because of the need to open a cranial window and also because it damages the brain parenchyma, which is not convenient for stroke patients.

The main alternative to this route of administration is the vascular route, either intra-arterial (i.a.) or intravenous (i.v.), which are currently the most used routes for cell delivery. I.v. injections are minimally invasive but cell tracking studies following this route have shown that most administered cells remain trapped in the lungs [104,105], liver [106], and spleen [107], indicating that a reduced number of cells reach the brain. On the contrary, i.a. administration is a promising strategy to direct the majority of injected cells to the brain, however, this is a risky administration route and the fate of injected cells following this route remains unknown due to high variation in the reported results [97].

Whether one route is more efficient than the other is not clear and depends on the cell type used. Thus, in some studies, it was found that the injection of neural progenitor cells by an i.a. route through the carotid artery presented a higher migration rate and a wider distribution pattern than i.v. administration. Nevertheless, the mortality rate for i.a. delivery was significantly higher (41%) than that for i.v. delivery (8%) [108]. However, in other studies with bone marrow stem cells and bone marrow mononuclear cells, there was no greater mortality or greater recovery of infarct volume of one route compared to the other [109,110].

The size of stem cells is also a critical parameter when passing through the lungs, and should be taken into consideration when deciding the best route of administration. For example, when using mesenchymal stem cells (MSCs), the majority of them become trapped in the lungs, while the neuronal stem cells (NSCs) have a two-fold higher pass-through rate [105]. 

**Figure 11:**
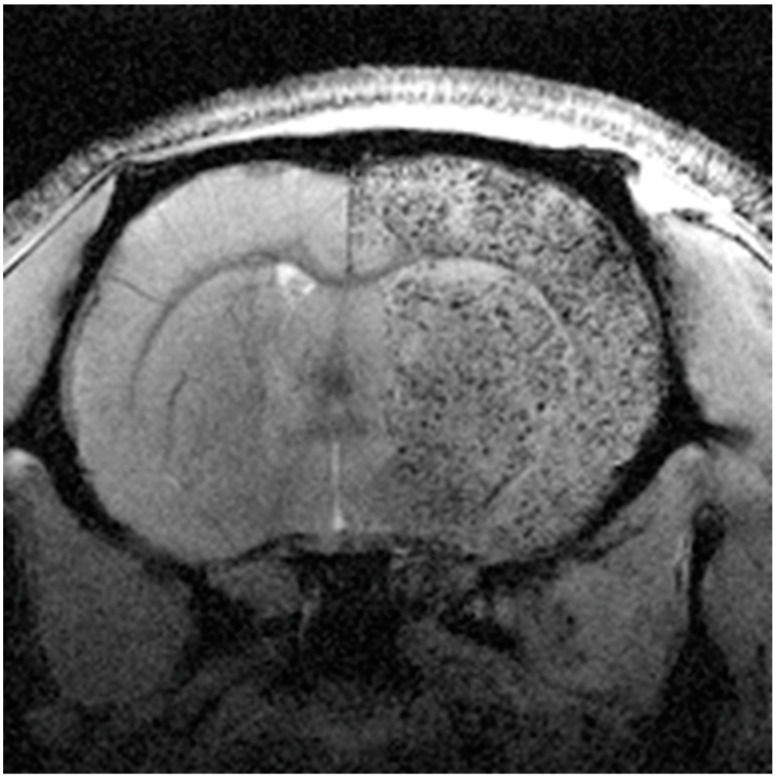
Magnetic resonance image (MRI) of a rat brain slice, 4 h after intraarterial administration of mesenchymal stem cells (MSCs) labeled with magnetic particles. Labeled cells can be observed as black punctate patterns in the right brain hemisphere. Reproduced with permission from [97], SpringerNature, 2017.

**Figure 12:**
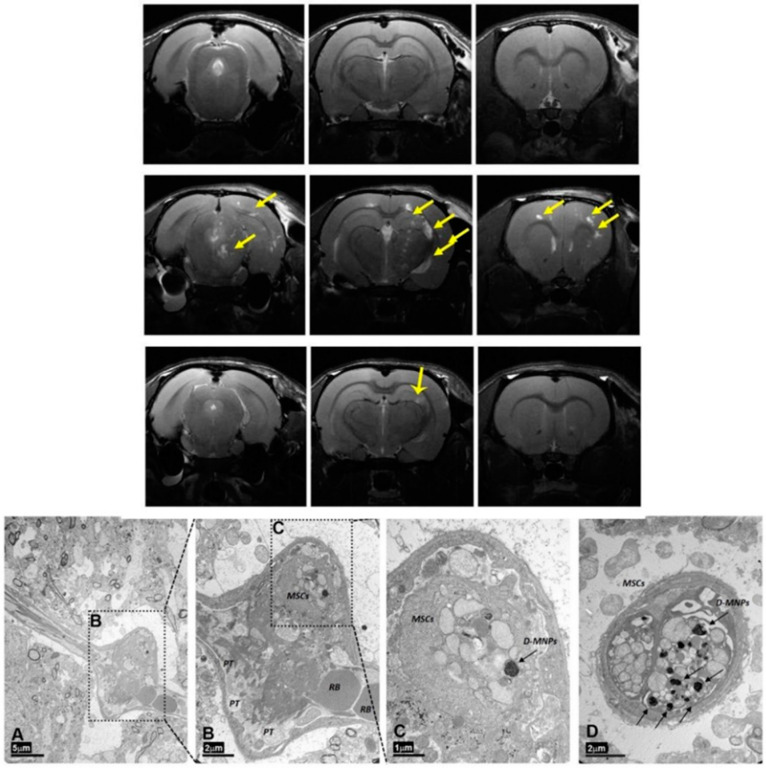
Upper figure: Multifocal ischemia is observed across the brain after administration of 1 × 10^6^ MSCs (indicated with yellow arrows). Lower figure. Electron transmission micrograph of the rat brain cortex 4 h after intra-arterial (i.a.) delivery of MSCs with magnetic particles, showing the arterial occlusion caused by the cell administration. (**A**) Dilated brain vessel surrounded by neuropils (scale bar 5 μm). (**B**) Magnification of vessel dilation in A. Red blood (RB) corpuscles are on the right, and platelets (PTs) and MSCs can be observed inside the vessel (scale bar 2 μm). (**C**) Magnification of the upper vessel expansion in B (scale bar 1 μm). (**D**) Longitudinal section of a brain vessel in which two labeled cells can be observed (scale bar 2 μm). Reproduced with permission from [97], SpringerNature, 2017.

To clarify the discrepancies in the best route for cell administration in stroke and using synthesized dextran-coated superparamagnetic NPs for MRI cell tracking, MSCs were investigated to determine if they are able to reach the brain following i.a. or i.v. administration. They were administered after transient cerebral ischemia in rats, and their therapeutic effects were evaluated for both routes of administration [97]. The findings from these studies show that MSCs were inside the brain tissue following i.a. but not i.v. administration in ischemic rats (Figure 11). However, the i.a. route increased the risk of cerebral lesions (microstrokes) (Figure 12) and did not improve functional recovery, while intravenous delivery produced functional recovery and was safe. This fact implies that the cell treatment benefits are not attributable to brain MCS engrafting after stroke [97]. Presumably, MSCs trapped in the lung would affect immune system function systemically, supporting the hypothesis that beneficial MSC effects after stroke can be achieved in the absence of significant MSC recruitment to the infarct region, and even overcome the associated risks of i.a. administration. The beneficial effect of i.v. MSC administration is mainly associated with the secretion of a large number of neuroprotective and neurotrophic factors that promote repair and recovery through numerous pathways [97].

### 5.3. Magnetic Vectorization 

Owing to the relevance of the route of administration and cell/dose on the efficacy of stem cell therapy, different strategies have emerged to improve cell delivery in target areas. In this regard, in order to increase the number of injected cells close to the ischemic lesion, magnetic vectorization of cells tagged with superparamagnetic NPs has been tested with successful results, showing that magnetic cell loading represents a safe and effective strategy for precise cell guidance into specific brain areas [111].

## 6. Discussion

Stroke is a disease that occurs unexpectedly and has a disastrous outcome. Approximately 15 million people experience a stroke episode every year worldwide, of which 33% are left with a permanent disability, whereas approximately 40% of cases result in death. Due to the high impact of the disease around the world, stroke is ranked as the second deadliest disease for individuals over 60 years of age. Despite the benefits of mechanical recanalization, rtPA thrombolysis remains the mainstay of acute stroke therapy in many hospitals, but its use is limited to a reduced number of patients, mainly due to the narrow therapeutic window and hemorrhagic complications. Many efforts have been made to investigate new protective drugs with successful results during preclinical analysis, but none of them have demonstrated benefits in clinical practice, and recovery cell therapies require additional clinical analysis and standardized protocols (for example, cell type, dose, or route of administration) before becoming a therapeutic resource for stroke patients. 

In the last decade, the development of nanomedicine has opened new and promising opportunities for the diagnosis and treatment of neurological pathologies, including stroke (Table 1). For instance, the use of NP contrast agents for stroke diagnosis or in vivo cell tracking has shown to be a powerful tool to study new aspects related to the imaging diagnosis or to evaluate the risks of cell therapies after administration; however, bioaccumulation or degradation are processes that are still not well known for many NPs and often depend on the material used; therefore, the use of NP contrast agents for clinical use requires further safety analysis. 

New advances in the field of nanotechnology have allowed the development of “smart” delivery nanoplatforms that have significantly improved the efficacy of thrombolytic treatments and protective drugs by reducing the side effects. The search for novel therapeutic strategies for stroke has increased significantly, and the combination of these therapies with efficient solutions provided by the field of nanotechnology would definitely help to find new effective therapeutic formulations to improve the outcomes of stroke patients. 

One of the major limitations of NPs as medicinal products is that after administration, these external “nanobodies” may activate the coagulation process with the risk of clotting. Moreover, NPs are rapidly sequestered from the bloodstream by circulating phagocytic cells and macrophages. This rapid sequestering represents one of the major challenges of nanoformulations for effective in vivo targeting of specific cells and tissues. The development of biomimetic nanosystems derived from cellular membranes has offered a novel solution to solve these limitations as they remain “invisible” to the phagocytic system, thereby increasing their free-life in blood circulation and improving the targeting efficiency. In the field of stroke, biomimetic nanosystems derived from cellular membranes, more specifically from platelets, have emerged as a promising strategy to improve the efficacy and safety of rtPA, with specific targeting of blood clots.

Finally, successful preclinical results with “smart” delivery nanoplatforms for stroke or other pathologies usually require scaled-up production for subsequent clinical validation. Risk analysis of scaled-up production should be taken into consideration to anticipate limitations such as synthesis issues, time consumption or poor batch consistency, and reproducibility, which could limit the translationality of the therapy. 

## Figures and Tables

**Table 1:** Summary of the most relevant nanosystems developed in the ischemia field.

**Diagnosis**			
**Imaging Tool**	**Type of Nanoparticle**	**Target Triggering**	**Reference**
MRI	MPIOs	VCAM-1	[18]
		ICAM-1	[19]
		P-selectin	[20]
		VCAM-1 and P-selectin	[21]
	Multimeric magnetite particles	Peptide to intracellular macrophage proteases	[23]
CT	Gold NPs	Fibrin	[22]
**Thrombolytic Treatment**			
**Type of Nanoparticle**		**Target Triggering**	**Reference**
Liposomes	Liposomes	Plasminogen	[33,34]
	Magnetoliposomes	Thermosensitive and magnetic guidance	[60]
Polymeric NPs	PLGA NPs coated with chitosan	Fibrin	[39,40]
	Polysaccharide-poly(isobutylcyanoacrylate) nanoparticles	P-selectin	[42]
	Layer-by-layer capsules	Glycoprotein (GP) IIb/IIIa Thrombin responsive	[43]
	Poly(N-isopropylacrylamide) nanogels	Fibrin	[44]
	Near-infrared fluorescent dye-conjugated boronated maltodextrin	Fibrin H_2_O_2_ responsive	[51]
	NIR fluorescent dye-conjugated boronate antioxidant polymers (fBAP) and fibrin-targeting lipopeptides	Fibrin H_2_O_2_ responsive	[52]
	Prodrug nanocarriers based on PEG and diosgenin derivatives	pH responsive	[53]
	Aggregates of multiple smaller NPs of PLGA	Shear stress responsive	[57]
	Layer-by-layer sub-micrometic nanocapsules with gelatin	Von Willebrand factor Ultrasounds	[65]
	Near-infrared fluorescent dye-conjugated boronated maltodextrin	Fibrin H_2_O_2_ responsive	[51]
Metal NPs	Magnetic rods	Magnetic guidance	[35]
	Fe_3_O_4_-based PLGA	Fibrin	[41]
	PLGA magnetic NPs	Fibrin Magnetic guidance	[46]
	Metal–organic framework-derived carbon nanostructures	GP IIb/IIIa Hyperthermia and ROS sensitive under NIR laser	[59]
	Chitosan nanocomposites with Fe_3_O_4_	Magnetic guidance	[61]
	Magnetic nanocubes within a deoxychitosan core	Magnetic guidance	[62]
	Magnetic Fe_3_O_4_ microrods	Magnetic guidance	[63]
Microbubbles		Fibrin Ultrasounds	[36]
		Fibrin Ultrasounds	[37]
Echogenic liposomes		Ultrasounds	[38]
		Ultrasounds	[64]
Biomimetic NPs	Red blood cells doped with NIR imaging agent		[49]
	Nanoplatelet with rtPA and a neuroprotectant (ZL006e)	Thrombin	[50]
	Platelet microparticle inspired	GP IIb/IIIa and P-selectin	[54]
**Neuroprotection**			
**Approach**	**Type of Nanoparticle**	**Target Triggering**	**Reference**
Inflammation	Exosomes loading curcumin	Integrin αvβ3	[71]
	Neutrophil-like cell membrane-coated mesoporous nanozyme	Inflamed endothelium	[72]
	Spheres and elliptical disks	ICAM-1	[79]
	Gold NPs with coumarin-PEG-thiol		[80]
	Sphere and rod NPs	VCAM-1	[81]
Oxidative stress	NPs of resveratrol		[85]
	Cerium oxide NPs		[86]
Glutamate excitotoxicity	Gold NPs with memantine		[88]
Combination with rtPA	Liposomes loading rtPA and fasudil		[90]
	Self-assembled antioxidant NPs with rtPA		[91]
**Neurorecovery**			
**Approach**	**Type of Nanoparticle**	**Target Triggering**	**Reference**
MRI cell tracking	Superparamagnetic iron oxide nanoparticles (SPIONs)	Cell tracking of mesenchymal stem cells (MSCs)	[97]
Magnetic vectorization	Superparamagnetic iron oxide nanoparticles (SPIONs)	Cell tracking of endothelial progenitor cells (EPCs) Magnetic guidance	[111]

Intercellular adhesion molecule 1 (ICAM-1); magnetic resonance imaging (MRI); near-infrared (NIR); nanoparticles (NPs); polyethylene glycol (PEG); poly lactic-co-glycolic acid (PLGA); reactive oxygen species (ROS); recombinant tissue plasminogen activator (rtPA); vascular cell adhesion molecule 1 (VCAM-1).

## Data Availability

Not applicable.
